# Mettl8 Regulates Hippocampal Neural Stem Cell Proliferation and Neurogenesis via mTOR/4E‐BP1 Signaling

**DOI:** 10.1002/cns.70953

**Published:** 2026-05-24

**Authors:** Jiamin Xu, Liping Xu, Yang Li, Xuejian Kong, Wenhui Ma, Linjie Xie, Ying Lyu, Aiguo Xuan

**Affiliations:** ^1^ School of Basic Medical Sciences, the Second Affiliated Hospital Guangzhou Medical University Guangzhou Guangdong Province China; ^2^ The Affiliated Traditional Chinese Medicine Hospital Guangzhou Medical University Guangzhou Guangdong Province China; ^3^ The Sixth Affiliated Hospital of Guangzhou Medical University Qingyuan People's Hospital Qingyuan Guangdong Province China; ^4^ Scientific Research Center of Guangzhou Medical University Guangzhou Guangdong Province China

**Keywords:** cell cycle arrest, hippocampal neurogenesis, methyltransferase‐like 8, mTOR inhibitors, neural stem cell homeostasis, neuronal differentiation, PI3K‐Akt pathway

## Abstract

**Aims:**

Hippocampal neural stem cells (NSCs) proliferation and differentiation are crucial for neuroregeneration, but their regulation remains unclear. This study investigates the role of the RNA methyltransferase Mettl8 in NSCs' fate determination.

**Materials and Methods:**

Stable Mettl8‐knockdown and overexpressing NSCs lines were generated via lentiviral transduction. Gene/protein expression was analyzed by qRT‐PCR, WB, and immunofluorescence. Proliferation and cell cycle were assessed using EdU assays and flow cytometry. Transcriptomic profiling was performed via RNA‐seq, and mechanistic validation was conducted with the mTOR inhibitor Rapamycin.

**Results:**

Mettl8 expression was higher in differentiated neurons and astrocytes than in NSCs. Functionally, Mettl8 knockdown induced G0/G1 arrest, reduced Cyclin E, and suppressed proliferation, while its overexpression promoted proliferation. Conversely, Mettl8 loss increased neuronal (βIII‐Tubulin) and astrocytic (GFAP) marker expression, whereas overexpression impaired differentiation. Mechanistically, Mettl8 negatively regulated the Akt/mTOR/4E‐BP1 pathway. Knockdown activated this pathway, and Rapamycin reversed the enhanced mTOR/4E‐BP1 phosphorylation and neuronal differentiation caused by Mettl8 loss.

**Conclusion:**

Mettl8 maintains NSCs' proliferative homeostasis and suppresses differentiation by fine‐tuning mTOR/4E‐BP1 signaling, revealing a potential therapeutic target for neuroregeneration.

## Graphical abstract

Mettl8‐mTOR/4E‐BP1 axis regulates hippocampal neurogenesis. Neural stem cells (NSCs) in the hippocampus are crucial for neuroregeneration. We found that the RNA methyltransferase METTL8 is highly expressed in neurons and astrocytes compared to NSCs. METTL8 knockdown in NSCs suppressed proliferation (reducing EdU+ cells and Cyclin E) but promoted neuronal and astrocytic differentiation. This effect was mediated by the excessive activation of the AKT–mTOR–4E‐BP1 pathway, which could be reversed by the mTOR inhibitor rapamycin. Thus, METTL8 maintains NSC proliferative homeostasis by fine‐tuning mTOR/4E‐BP1 signaling.

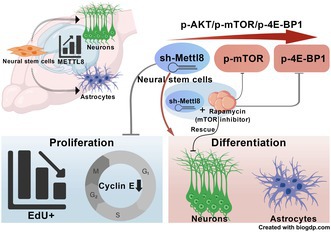

## 1 Introduction

The subgranular zone (SGZ) of the hippocampal dentate gyrus contains neural stem cells (NSCs) whose self‐renewal and differentiation are regulated by specialized microenvironments. Symmetric NSCs division maintains the stem cell pool, while asymmetric division produces neural progenitors that integrate into hippocampal circuits and mature into functional granule neurons essential for learning and memory [1, 2]. Impaired hippocampal neurogenesis is linked to Alzheimer's disease (AD) and major depression [3, 4, 5]. Although Notch and Wnt signaling are known regulators [6, 7], critical gaps remain: (1) undefined spatiotemporal thresholds of signaling components; (2) poorly understood pathway crosstalk; and (3) unidentified upstream regulators of key hubs like mTOR [8], hindering targeted regenerative therapies.

Methyltransferase‐like 8 (METTL8) is an RNA‐modifying enzyme with a conserved methyltransferase domain [9, 10] that produces functionally distinct isoforms, with the mitochondrial isoform Mettl8‐Iso1 known to modify mitochondrial tRNA m [3] C32 [11], while the functional roles of nuclear isoforms (Iso3/Iso4) remain largely uncharacterized [12]. Key evidence from other contexts suggests regulatory roles: Embryonic brain‐enriched expression [9]; cell fate modulation via STAT3/JNK [13]; myofiber transformation via miR‐208b suppression [14]; and clinical correlation with pancreatic cancer survival [15]. However, METTL8's expression pattern and functional role in neural regeneration, particularly in hippocampal neurogenesis, remain unknown.

The mTOR pathway regulates neurogenesis via mTORC1 (cell growth) and mTORC2 (cell survival) [16]. while mTORC1 activation promotes cortical progenitor proliferation [17], its dysregulation impairs ventricular zone stem cell renewal [18]. Downstream 4EBP1 critically controls translational initiation during neuronal differentiation [17, 19]. Current limitations include unidentified mTOR upstream regulators and unvalidated METTL8‐AKT interactions in neural contexts [20]. Establishing a METTL8‐mTOR/Eif4ebp1 axis represents a pivotal opportunity.

This study uses a comprehensive approach: Defining the expression pattern of METTL8 across neural lineages in the hippocampal SGZ, functional validation via lentiviral knockdown/overexpression, and mechanistic dissection via RNA‐seq and mTOR target validation with rapamycin rescue. By elucidating how METTL8 regulates neurogenesis via the mTOR/Eif4ebp1 axis, we provide a potential RNA‐targeted therapeutic perspective for neurodegenerative diseases.

## 2 Methods

### 2.1 Experimental Animals

Male C57BL/6 mice (8‐week‐old, 20–25 g) were purchased from Guangdong Medical Laboratory Animal Center (License No. SCXK 2022–0002). Mice were housed in specific pathogen‐free (SPF) conditions at Guangzhou Medical University Animal Center under controlled environment (22°C ± 2°C, 50% ± 10% humidity, 12/12 h light–dark cycle) with ad libitum access to food and water. All animal experiments were approved by the Institutional Animal Care and Use Committee of Guangzhou Medical University (S2022‐245) and were conducted in accordance with the national guidelines for animal welfare.

Euthanasia and tissue collection: Mice were euthanized by overdose anesthesia to ensure minimal suffering, in accordance with ethical guidelines. Specifically, mice were deeply anesthetized via intraperitoneal injection of pentobarbital sodium (at a dose of 150 mg/kg). Upon reaching a surgical plane of anesthesia (as indicated by the absence of pedal reflex), transcardial perfusion was immediately performed with 4% paraformaldehyde (PFA) in phosphate buffer. Following perfusion, brains were harvested, post‐fixed, cryoprotected, and sectioned for staining as described below.

### 2.2 Cell Culture and Lentiviral Stable Sell Line Construction

Adult rat hippocampal NSCs (Merck Millipore, Cat# SCR022) were maintained in 50 μg/mL poly‐L‐ornithine (Sigma, Cat# P4832) and 10 μg/mL synthetic laminin peptide (Merck Millipore, Cat# SCR127) coated dishes. Cells were cultured in the neural stem cell basal medium (Merck Millipore, Cat# SCM003) under 37°C/5% CO₂ conditions. For proliferation, the basal medium was enriched with 20 ng/mL EGF (PeproTech, Cat# AF‐100‐15) and 20 ng/mL bFGF (PeproTech, Cat# 100‐18B‐500). As required, the rapamycin (MedChemExpress, Cat# AY‐22989) was added to the proliferation medium at different concentrations to attain the optimum. For neuronal and astrocytic differentiation, the basal medium was enriched with 10 μM retinoic acid (RA, MedChemExpress, Cat# HY‐14649) and 20 ng/mL IL‐6 (MedChemExpress, Cat# HY‐P7063), respectively. The cell differentiation was kept in the corresponding medium for 5–7 days with or without rapamycin. Standard procedures involved: Thawing (37°C water bath → 1,000 × g/5 min centrifugation → resuspension), passaging (accutase digestion, 3 min, Sigma, Cat# A6964), and cryopreservation (90% cell medium/10% DMSO, MP, Cat# 196055). Cells were seeded at optimized densities: 2.5 × 10^5^ cells/6‐cm dish (protein/RNA extraction), 2 × 10^4^ cells/24‐well (immunofluorescence), and 5 × 10^3^ cells/96‐well (EdU assay), with half‐medium changes every 24–48 h.

Lentiviral vectors for sh‐Mettl8 (short hairpin‐Mettl8), OE‐Mettl8 (overexpress‐Mettl8), and negative control were packaged by OBiO Technology (Shanghai, China). For stable line generation, NSCs were seeded in 24‐well plates (1 × 10^4^ cells/well) and infected for 12 to 16 h with 5 μg/mL polybrene (Sigma, Cat# H9268). When the cell growth density is approximately 80%, the puromycin (1 μg/mL, TIENGEN BIOTECH, Cat# P7255) selection commenced, lasting 5 days until non‐infected cells completely died. Surviving cells (lentiviral stable knockdown and overexpression lines) were expanded and collected for subsequent cell experiments.

**FIGURE 1:**
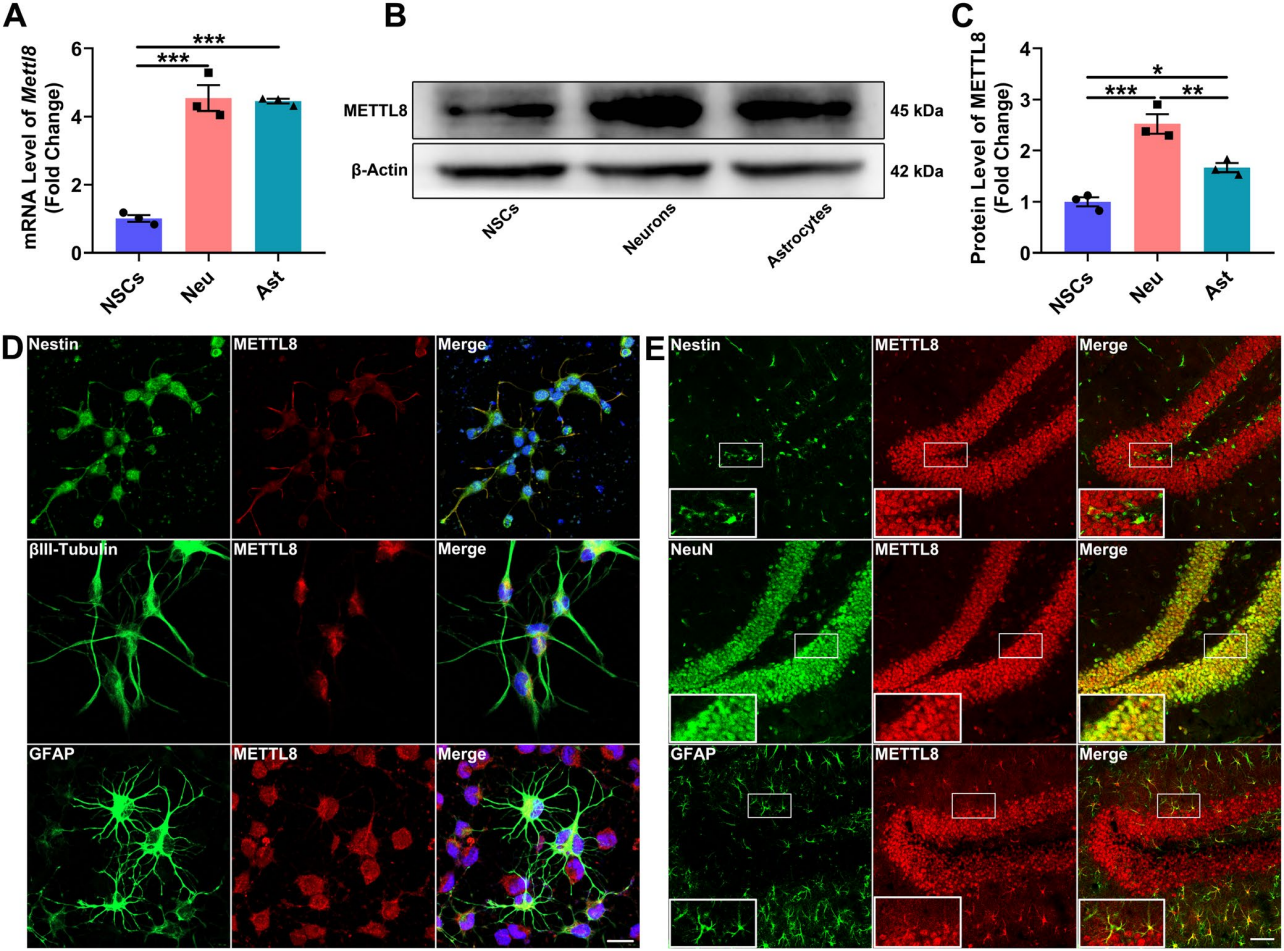
Preferential enrichment of Mettl8 in neurons. (A) qRT‐PCR analysis of Mettl8 mRNA levels in NSCs, neurons (Neu), and astrocytes (Ast) derived from the rat NSCs line. (B) Representative WB of METTL8 protein expression. β‐Actin served as a loading control. (C) Quantification of (B). (D) Immunofluorescence co‐staining of METTL8 (red) with Nestin (NSCs, green), βIII‐Tubulin (neurons, green), or GFAP (astrocytes, green). Nuclei were counterstained with DAPI (blue). Scale bars: 20 μm. (E) Immunohistochemistry of the mouse hippocampus showing METTL8 signals. Scale bars: 50 μm. *P < 0.05, **p < 0.01, ***p < 0.001. (Data in A–D are from rat cells; data in E are from mouse tissue. Data are presented as mean ± SEM from *n* = 3 independent biological replicates. Statistical significance was determined by one‐way ANOVA.)

**FIGURE 2:**
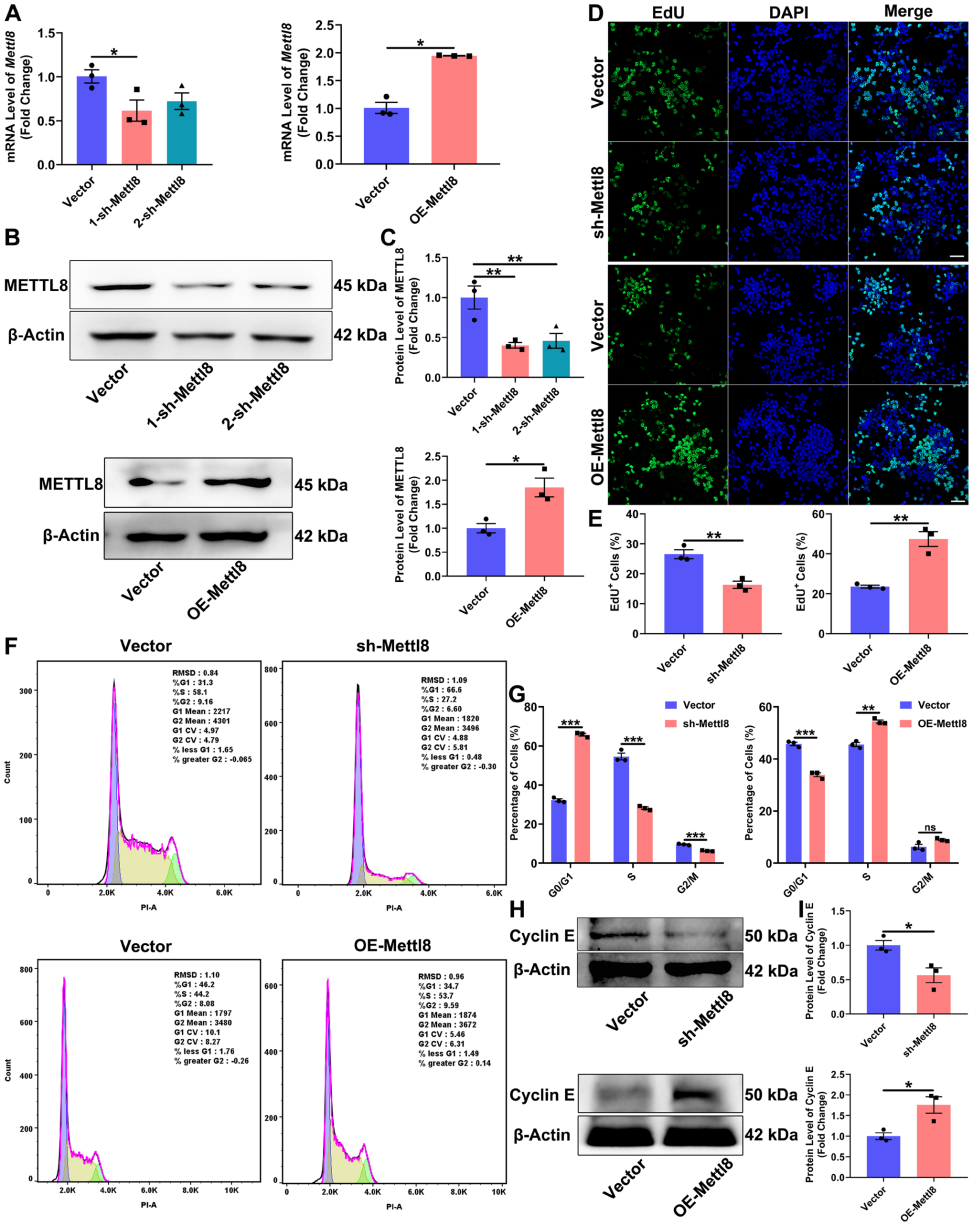
Mettl8 Promotes proliferation of rat NSCs. (A) Mettl8 mRNA levels after knockdown (sh‐Mettl8) or overexpression (OE‐Mettl8). (B, C) Representative WB images and quantification of METTL8 protein. β‐Actin served as a loading control. (D) Representative images of EdU+ cells (green). Nuclei were counterstained with DAPI (blue). Scale bar: 50 μm. (E) Quantification of EdU+ cells. (F, G) Cell cycle distribution analyzed by flow cytometry. (H, I) Representative WB images and quantification of Cyclin E protein. β‐Actin served as a loading control. *p < 0.05, **p < 0.01, ***p < 0.001. (Data are presented as mean ± SEM from *n* = 3 independent biological replicates. Inter‐group differences of three groups were analyzed by one‐way ANOVA for A and C; inter‐group comparisons of two groups were performed using an independent samples *t*‐test.)

**FIGURE 3:**
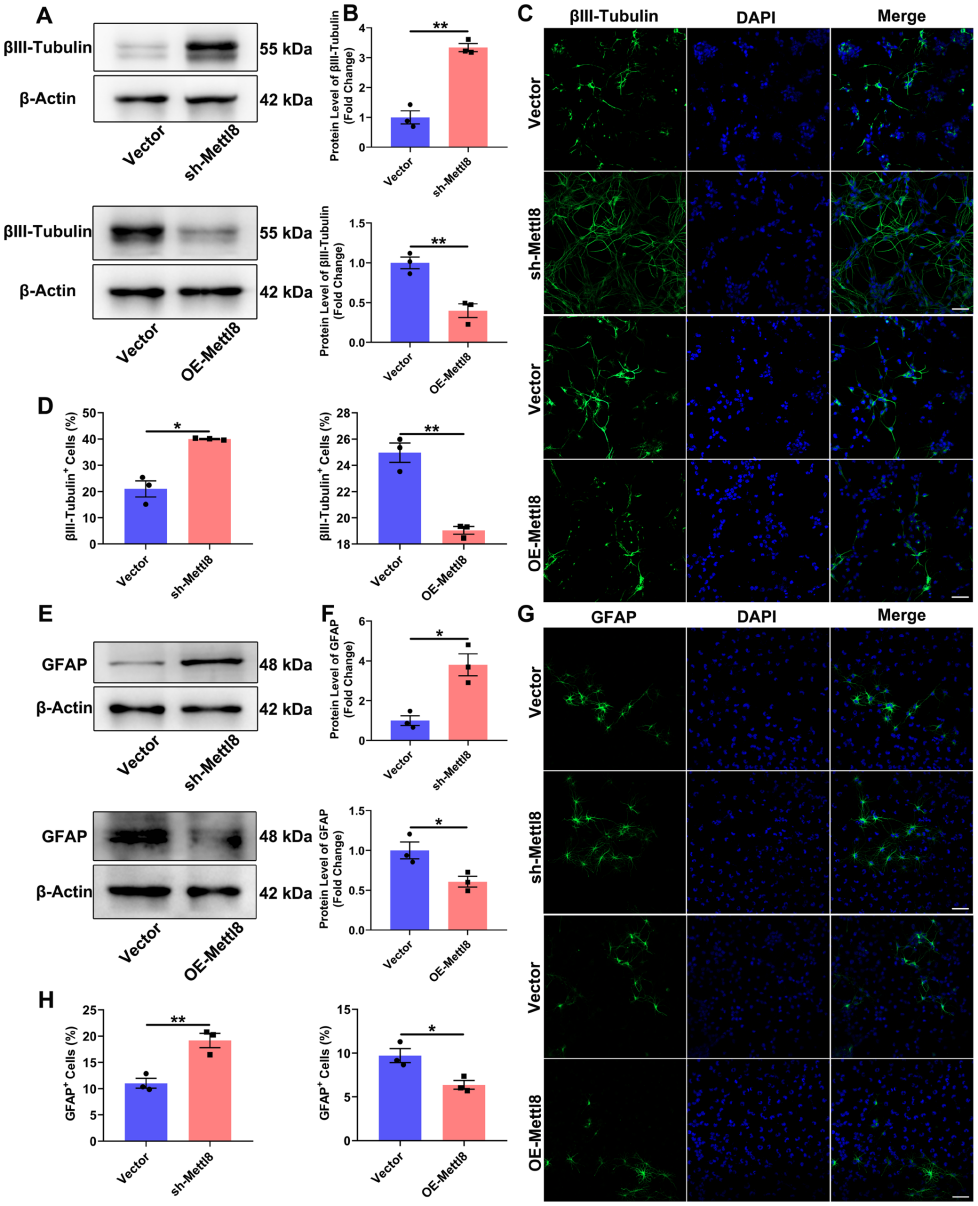
Mettl8 Suppresses differentiation of rat NSCs. (A, B) WB analysis and quantification of the neuronal marker βIII‐Tubulin. β‐Actin served as a loading control. (C, D) βIII‐Tubulin immunofluorescence (green) and quantification. Nuclei were counterstained with DAPI (blue). Scale bar: 50 μm. (E, F) WB analysis and quantification of the astrocytic marker GFAP. β‐Actin served as a loading control. (G, H) GFAP immunofluorescence (green) and quantification. Nuclei were counterstained with DAPI (blue). Scale bar: 50 μm. *P < 0.05, **p < 0.01, ***p < 0.001. (Data are presented as mean ± SEM from *n* = 3 independent biological replicates. Inter‐group comparisons were performed using an independent samples *t*‐test.)

**FIGURE 4:**
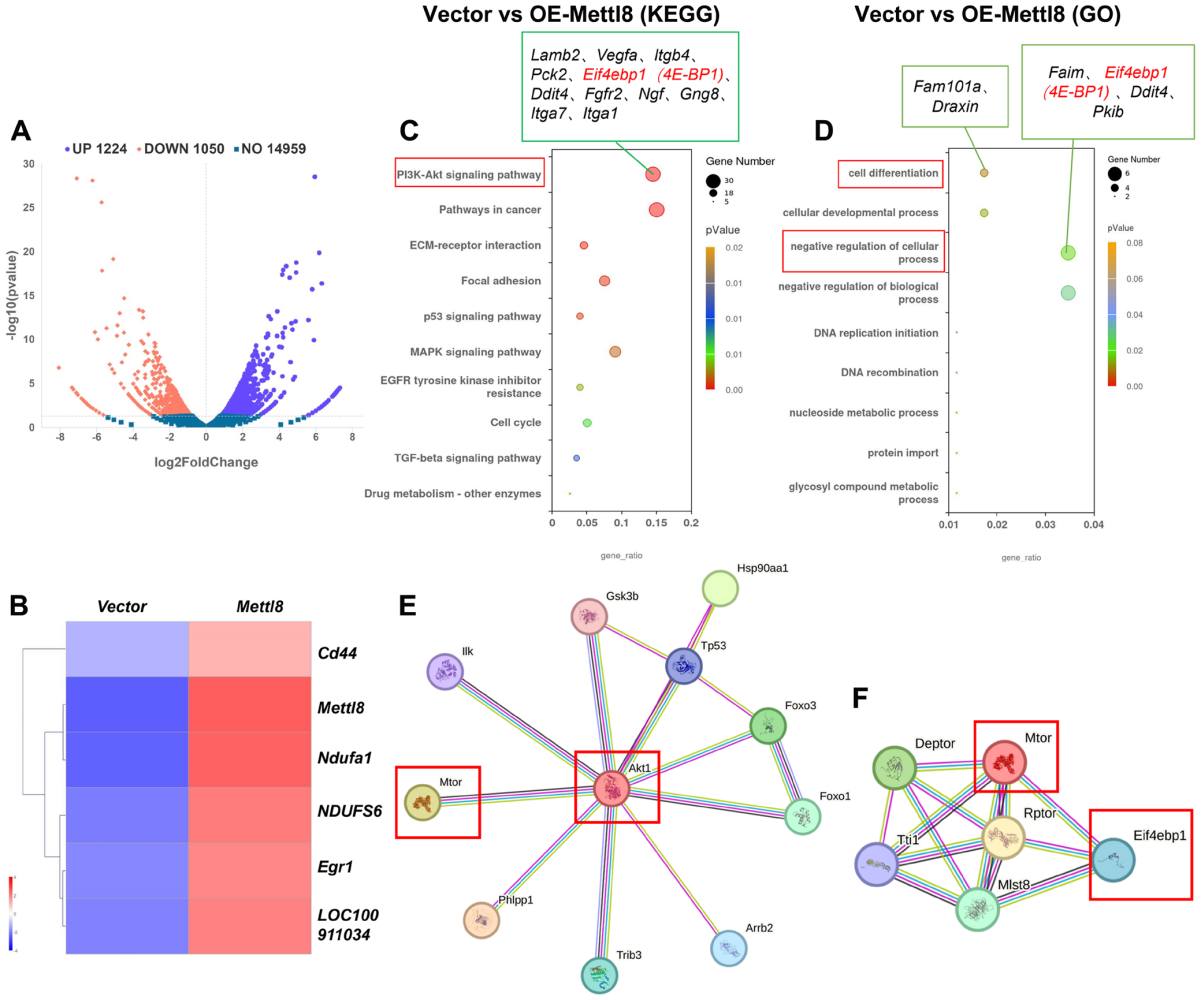
Transcriptomic profiling of Mettl8‐Overexpressing rat hippocampal NSCs. (A) Volcano plot of differentially expressed genes (DEGs, FDR < 0.05). (B) Clustering heatmap of DEGs. (C) Top 10 enriched KEGG pathways. (D) Gene Ontology (GO) term enrichment analysis. (E, F) Protein–protein interaction (PPI) networks of Akt and mTOR, respectively. (Transcriptomic analysis was performed on RNA extracted from rat NSCs). Data are from *n* = 3 independent biological replicates per group (Vector and OE‐Mettl8).

**FIGURE 5:**
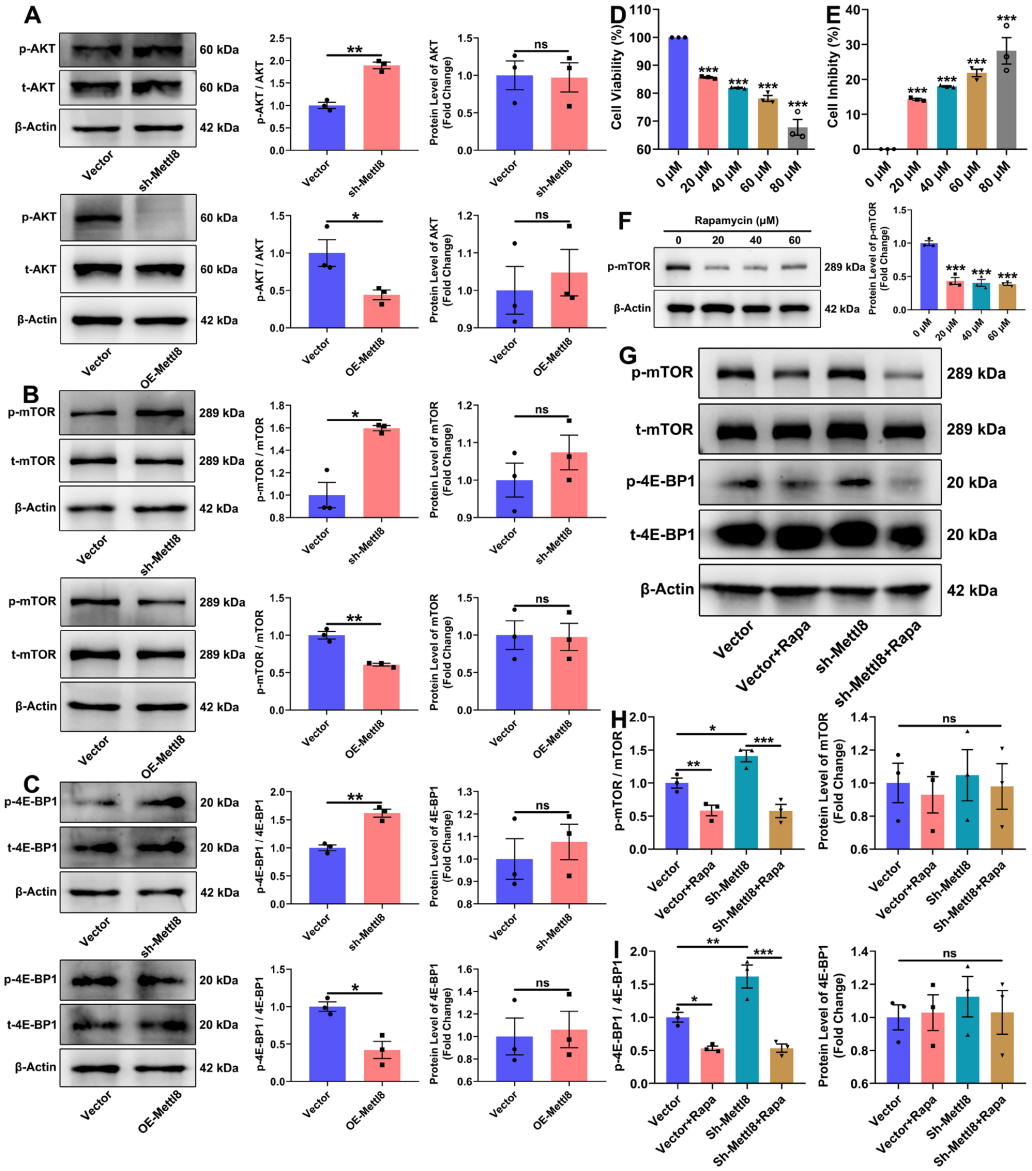
Mettl8 Regulates the function of rat NSCs via mTOR signaling. (A–C) WB analysis and quantification of p‐AKT/AKT, p‐mTOR/mTOR, and p‐4E‐BP1/4E‐BP1 ratios. β‐Actin served as a loading control. (D, E) Dose‐dependent effects of rapamycin on cell viability (CCK‐8 assay) (***p < 0.001 vs. 0 μM). (F) WB showing p‐mTOR levels after rapamycin treatment. β‐Actin served as a loading control. (G–I) WB analysis and quantification of indicated proteins in rescue experiments. β‐Actin served as a loading control. *p < 0.05, **p < 0.01, ***p < 0.001. (Data are presented as mean ± SEM from *n* = 3 independent biological replicates. Inter‐group comparisons were performed using an independent samples *t*‐test for A–C; inter‐group differences were analyzed by one‐way ANOVA for D–I).

**FIGURE 6:**
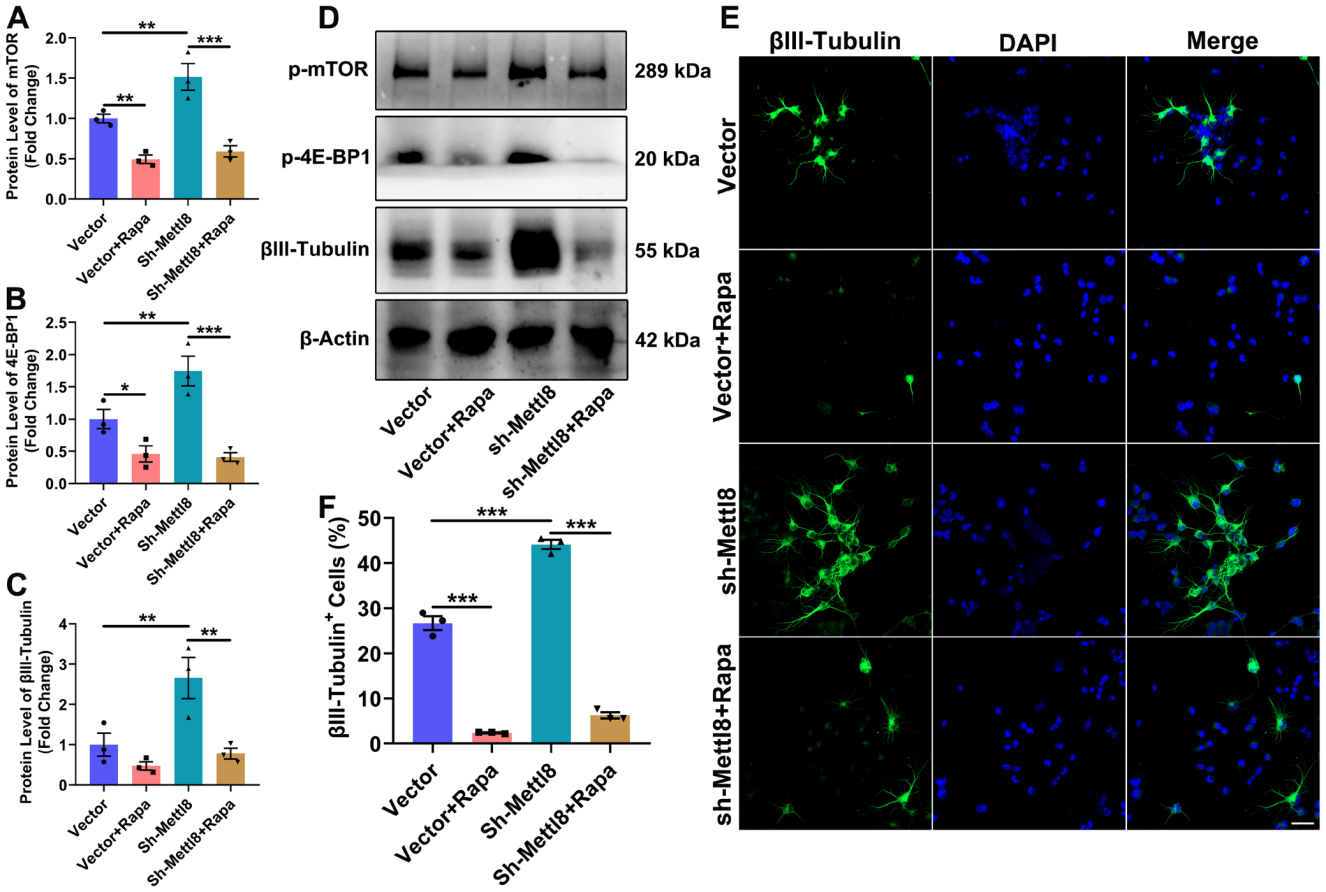
Mtor signaling mediates Mettl8‐regulated neuronal differentiation in rat NSCs. (A–D) WB analysis and quantification of p‐mTOR, p‐4E‐BP1, and the neuronal marker βIII‐Tubulin. β‐Actin served as a loading control. (E, F) βIII‐Tubulin immunofluorescence (green) and quantification. Nuclei were counterstained with DAPI (blue). Scale bar: 50 μm. *P < 0.05, **p < 0.01, ***p < 0.001. (Data are presented as mean ± SEM from *n* = 3 independent biological replicates. Inter‐group differences were analyzed by one‐way ANOVA.)

Rationale for species selection: The use of different species across experimental approaches was intentional. Mouse hippocampal tissue was employed for in vivo immunohistochemical validation (Section 2.1, Figure 1E) due to the extensive availability of well‐characterized anatomical databases and high‐quality commercial antibodies validated for mouse tissue. All functional and mechanistic studies were conducted using a well‐established rat hippocampal NSCs line (Sections 2.2, 2.8, Figures 2, 3, 4, 5, 6), which provides a robust, homogeneous, and genetically tractable model for in vitro manipulation. Consequently, all subsequent molecular analyses (qRT‐PCR, WB, RNA‐seq) were performed on this rat‐derived cellular material, and RNA sequencing data were aligned to the rat genome ( *Rattus norvegicus* Rnor_6.0) as specified in Section 2.7.

### 2.3 Quantitative Real‐Time PCR (qRT‐PCR)

Total RNA was extracted from NSCs using a TRIzol reagent (Takara, Cat# 9109). After PBS washing, cells were lysed with 1 mL TRIzol per dish (5 min, ice). Following chloroform extraction (200 μL) and centrifugation (12,000 × g, 15 min, 4°C), RNA was precipitated with isopropanol and washed with 75% ethanol (prepared in DEPC‐treated water). The RNA concentration was quantified by Nanodrop 2000 (Thermo). Genomic DNA was removed, and cDNA synthesis was performed using a kit (Takara, Cat# RR047A). The qRT‐PCR reactions used the SYBR Premix Ex Taq (Takara, Cat# RR820A) on Bio‐Rad CFX96 (Bio‐Rad). Primer sequences were as follows: *Mettl8* forward: 5′‐AGTCTCAGGTTGTCAGTTGTCT‐3′; *Mettl8* reverse: 5′‐AAGAGCAGTCAAGAGCAGAGT‐3′. *GAPDH* forward: 5′‐GCGAGATCCCGCTAACATCA‐3′; *GAPDH* reverse: 5′‐CTCGTGGTTCACACCCATCA‐3′ (Sangon Biotech). All reactions included triplicate technical replicates, with relative quantification using the 2^‒ΔΔCt^ method.

### 2.4 Western Blotting (WB)

Proteins were extracted using RIPA buffer containing PMSF and protease inhibitor cocktail (RIPA:PMSF:inhibitor = 1000:10:1, KEYGEN BIOTECH, Cat# KGP702, KGP610, KGB5101‐100). After PBS washing, cells were scraped and sonicated on ice (30% amplitude, 2 s on/2 s off, 5 cycles). Lysates were centrifuged (12,000 × g, 10 min, 4°C) following 30‐min incubation. The protein concentration was determined by Pierce BCA Protein Assay Kit (Thermo Fisher Scientific, Cat# 23209) with bovine serum albumin standards. Samples were adjusted to a uniform protein concentration using PBS and 4× LDS buffer, denatured at 100°C for 10 min, and stored at −80°C. The SDS‐PAGE was performed using kits (Beyotime, Cat# P0012A) with gel concentrations optimized for target proteins. Samples were separated at 60 V (stacking) and 120 V (resolving gel; bromophenol blue tracking). Proteins were transferred to methanol‐activated PVDF membranes (Millipore, Cat# ISEQ00010). Membranes were blocked with 5% BSA/TBST (2 h, room temperature, RT), incubated with primary antibodies (see Table 1 for details) overnight (4°C), washed (TBST, 3 × 10 min), and probed with HRP‐conjugated secondary antibodies (see Table 1) for 1 h (RT). Signals were detected by an ECL substrate (Millipore, Cat# WBKLS0500). The experiments were performed in triplicate.

**TABLE 1:** Primary and secondary antibodies used in this study.

Antibody	Company	Code	Dilution
Anti‐β‐Actin	Cell Signaling Technology	4970S	WB‐1:2000
Anti‐METTL8	Biorbyt	orb451475	WB‐1:1000; IF‐1:200
Anti‐Cyclin E	Abcam	ab317436	WB‐1:1000
Anti‐βIII‐Tubulin	Abcam	ab78078	WB‐1:1000; IF‐1:400
Anti‐GFAP	Cell Signaling Technology	3670S	WB‐1:1000; IF‐1:400
Anti‐p‐AKT	Cell Signaling Technology	4060S	WB‐1:1000
Anti‐AKT	Cell Signaling Technology	4691	WB‐1:1000
Anti‐p‐mTOR	Cell Signaling Technology	5536S	WB‐1:1000
Anti‐mTOR	Cell Signaling Technology	2983 T	WB‐1:1000
Anti‐p‐4E‐BP1	Cell Signaling Technology	2855S	WB‐1:1000
Anti‐4E‐BP1	Cell Signaling Technology	9452	WB‐1:1000
Anti‐Nestin	NOVUSBIO	NB100‐1604	IF‐1:400
Anti‐NeuN	Cell Signaling Technology	94403S	IF‐1:400
Anti‐rabbit HRP‐linked IgG	Cell Signaling Technology	7074	WB‐1:2000
Anti‐mouse HRP‐linked IgG	Cell Signaling Technology	7076	WB‐1:2000
Anti‐rabbit, Alexa Fluor 594	Invitrogen	A21207	IF‐1:500
Anti‐mouse, Alexa Fluor 488	Invitrogen	A21202	IF‐1:500
Anti‐chicken, Alexa Fluor 488	Invitrogen	A11039	IF‐1:500

### 2.5 Immunofluorescence (IF) Staining

Cellular staining: Cells on coverslips (24‐well plate, 80% cell density) were fixed with 4% paraformaldehyde (PFA, 20 min, RT), permeabilized with 0.3% Triton X‐100 (20 min, Beyotime, Cat# ST795), and blocked with 5% BSA/PBS (1–2 h). Primary antibodies (detailed in Table 1) were incubated overnight at 4°C: Mettl8 co‐stained with βIII‐Tubulin, NeuN, GFAP, or Nestin. Alexa Fluor‐conjugated secondary antibodies (Table 1) incubated for 1 h (RT, dark), followed by DAPI (10 min, KEYGEN BIOTECH, Cat# KGF0281‐0282). Cells were imaged by a laser scanning confocal microscope (Zeiss).

Tissue staining: Mice were perfused with 4% PFA. Brains were post‐fixed overnight and dehydrated in 20%–30% sucrose. The coronal sections (30 μm) were cut with a freezing microtome (Leica). Sections were blocked and permeabilized in 0.3% Triton X‐100/5% BSA (1 h). The antibody incubation was identical to the cellular protocol.

EdU assay: Cells were treated with a kFluor488‐EdU cell proliferation assay kit (Beyotime, Cat# C0071S), following the manufacturer's protocol to get cell samples. Other steps, like fixation, blocking, incubation with antibodies, and counterstaining, were referred to as preceding steps.

The experiments were independently repeated three times.

### 2.6 Flow Cytometric Cell Cycle Analysis

Proliferating NSCs were detached with Accutase (Sigma, Cat# A6964) at 37°C for 3 min. Cells were resuspended in PBS containing 1% FBS, centrifuged (200 × g, 5 min), and fixed in ice‐cold 75% ethanol (added dropwise) overnight at 4°C. After washing with cold PBS (200 × g, 3 min), cells were stained with PI working solution from Cell Cycle Analysis Kit (Beyotime, Cat# C2015S) for 30 min at 37°C in the dark. Samples were filtered through 35 μm meshes and analyzed on the CytoFLEX (BD FACSAria) with parameters: FSC 80 V, SSC 205 V, PI 240 V (linear mode). Data acquisition included: (1) FSC‐A/SSC‐A dot plot (cell population gating), (2) PI‐W/PI‐A plot (doublet exclusion), (3) PI‐A histogram (cycle phase quantification). A minimum of 20,000 single‐cell events were recorded per sample. Cell cycle distribution (G0/G1, S, G2/M phases) was quantified using the FlowJo software.

### 2.7 Transcriptome Sequencing (Seq) and Bioinformatics Analysis

To investigate the impact of *Mettl8* overexpression on the transcriptome of NSCs, RNA‐seq was performed on control (Vector) and Mettl8‐overexpressing (OE‐Mettl8) groups. Total RNA was extracted using a TRIzol reagent (Takara, Cat# 9109). After quality control using an Agilent 2100 bioanalyzer, library preparation and sequencing were conducted by the Novogene Co. Ltd. Briefly, mRNA was enriched from the total RNA using oligo(dT) magnetic beads, followed by the ribosomal RNA depletion. The enriched mRNA was fragmented and used for first‐ and second‐strand cDNA synthesis. The purified cDNA was end‐repaired, adenylated, ligated with sequencing adaptors, and PCR‐amplified to construct the final sequencing libraries. The library quality was assessed using a Qubit 2.0 Fluorometer (for concentration), an Agilent 2100 bioanalyzer (for size distribution), and qRT‐PCR (for amplification efficiency). Qualified libraries were sequenced on an Illumina HiSeq 3000 platform. Raw sequencing reads were subjected to strict quality control. Adapter sequences, reads containing ambiguous bases (N), and low‐quality reads were filtered out to obtain high‐quality clean reads. These clean reads were then aligned to the reference genome (ensembl_rattus_norvegicus_rnor_6_0_gca_000001895_4) using the HISAT2 software to obtain their mapping positions. The differential expression analysis between groups was carried out using the DESeq2 package. Differentially expressed genes (DEGs) were identified with the thresholds of |log2(Fold Change)| > 1 and an adjusted *p*‐value (FDR, padj) < 0.05. The identified DEGs were subsequently subjected to Gene Ontology (GO) functional enrichment analysis, Kyoto Encyclopedia of Genes and Genomes (KEGG) pathway enrichment analysis, and protein–protein interaction (PPI) network analysis using the STRING database.

### 2.8 Cell Proliferation and Cytotoxicity Assay (CCK‐8)

Cells were seeded in 96‐well plates at 2000 cells/well (proliferation) or 5000 cells/well (cytotoxicity) with 100 μL complete medium. For cytotoxicity assays, gradient concentrations of rapamycin (0, 20, 40, 60, 80 μM) were added. After incubation (24 h), 10 μL CCK‐8 reagent (Beyotime, Cat# C0037) was added per well. Following 4 h incubation at 37°C/0.5% CO_2_ (optimal time determined by pre‐testing 0.5/1/2/4 h), absorbance was measured at 450 nm wavelength (Tecan Spark). Based on a systematic screening of both CCK‐8 and mTOR pathway inhibitory efficacy (WB for p‐mTOR) across a gradient (0–80 μM), 20 μM rapamycin was selected as the optimal working concentration for our rat NSCs line. This concentration was determined to achieve near‐complete inhibition of p‐mTOR in our NSCs culture system (Figure 5F) while maintaining cell viability above 85% (Figure 5D,E), thereby balancing efficacy and minimal cytotoxicity.

### 2.9 Statistical Analysis

Data were derived from ≥ 3 independent biological replicates with triplicate technical repeats per group. Results are presented as mean ± SEM. Analyses were performed in SPSS16.0 following a standardized protocol: Normality → homoscedasticity → two‐group comparisons: Independent samples *t*‐test/multiple‐group comparisons: One‐way ANOVA → significance threshold: *p* < 0.05.

## 3 Results

### 3.1 Differential Expression of Mettl8 Across Neural Cell Lineages

Mettl8 expression was upregulated in differentiated neural cells. Unless otherwise specified, all in vitro cellular assays were performed using a rat hippocampal NSCs line. Compared to NSCs, both mRNA (Figure 1A) and protein (Figure 1B,C) levels of Mettl8 were significantly higher in neurons and astrocytes (*p* < 0.05), with the most prominent upregulation observed in neurons. Immunofluorescence co‐staining revealed more intense METTL8 signals in βIII‐Tubulin+ neurons and GFAP+ astrocytes than in Nestin+ NSCs (Figure 1D). This stage‐specific enrichment was further validated in vivo, as stronger METTL8 signals were detected in hippocampal neurons and astrocytes compared to NSCs in mouse brain sections (Figure 1E).

### 3.2 Mettl8 Promotes the Proliferation of NSCs

Stable Mettl8‐knockdown (sh‐Mettl8) and overexpression (OE‐Mettl8) rat NSCs lines were established (Figure 2A–C). The EdU assay showed that sh‐Mettl8 significantly reduced the percentage of proliferating cells, whereas OE‐Mettl8 increased it by approximately 2‐fold (*p* < 0.01; Figure 2D,E). Flow cytometry analysis revealed that Mettl8 knockdown induced G0/G1 phase arrest and decreased S‐phase population, along with a reduction in Cyclin E protein. Conversely, Mettl8 overexpression accelerated G1/S transition and increased Cyclin E levels (Figure 2F–I). These findings indicate that Mettl8 drives NSCs’ proliferation by facilitating the G1/S transition and positively regulating Cyclin E.

### 3.3 Mettl8 Suppresses the Neuronal and Glial Differentiation of NSCs

To investigate the role of Mettl8 in rat hippocampal NSCs differentiation, we induced lineage specification in Mettl8‐knockdown (sh‐Mettl8) and overexpression (OE‐Mettl8) cells. Bidirectional validation by western blot and immunofluorescence revealed that sh‐Mettl8 significantly upregulated the neuronal marker βIII‐Tubulin (3.3‐fold increase in protein, *p* < 0.01; 19.7% ± 5.5% more positive cells, *p* < 0.05; Figure 3A–D) and the astrocytic marker GFAP (3.8‐fold increase in protein, *p* < 0.05; 8.3% ± 1.6% more positive cells, *p* < 0.01; Figure 3E–H). Conversely, OE‐Mettl8 impaired differentiation capacity, reducing βIII‐Tubulin protein to 39.9% of control (*p* < 0.01) with 5.9% ± 1.7% fewer βIII‐Tubulin+ cells (*p* < 0.01), and decreasing GFAP protein to 60.7% of control (*p* < 0.05) with a 3.3% ± 0.7% reduction in GFAP+ cells (*p* < 0.05) (Figure 3A–H). These findings demonstrate that Mettl8 bidirectionally regulates differentiation markers, suppressing NSCs’ commitment to both neuronal and astrocytic lineages.

### 3.4 mTOR/4E‐BP1 Are Downstream Effectors of Mettl8

To elucidate the mechanism of Mettl8, we performed RNA sequencing on Mettl8‐overexpressing rat NSCs (Figure 4A,B). KEGG and GO analyses collectively indicated significant enrichment of differentially expressed genes in pathways including PI3K‐Akt signaling. Notably, Eif4ebp1 (encoding 4E‐BP1) emerged as a key overlapping gene (Figure 4C,D). Protein interaction network analysis further suggested that mTOR interacts with Akt and phosphorylates 4E‐BP1 (Figure 4E,F), leading us to hypothesize that Mettl8 might regulate 4E‐BP1 via the Akt/mTOR axis.

WB validation confirmed that Mettl8 knockdown (sh‐Mettl8) significantly increased the phosphorylation levels of p‐AKT, p‐mTOR, and p‐4E‐BP1, while its overexpression (OE‐Mettl8) had the opposite effect, without altering total protein levels (Figure 5A–C). This establishes Mettl8 as a negative regulator of the Akt/mTOR/4E‐BP1 phosphorylation cascade. The concomitant cell cycle arrest upon Mettl8 knockdown (Figure 2) suggests that the functional outcome of this mTOR activation is context‐dependent, steering cells away from proliferation. To investigate whether mTOR activation mediates the effects of Mettl8 loss, we employed the mTOR‐specific inhibitor rapamycin. Based on systematic screening for optimal efficacy and minimal cytotoxicity in our cell model (see Methods), 20 μM rapamycin was selected for subsequent rescue experiments (Figure 5D–F). Rapamycin treatment reduced p‐mTOR and p‐4E‐BP1 levels in control cells. Crucially, the elevated p‐mTOR and p‐4E‐BP1 levels induced by sh‐Mettl8 were completely reversed by co‐treatment with rapamycin (Figure 5G–I). This pharmacological rescue demonstrates that mTOR activation upon Mettl8 knockdown is reversible.

### 3.5 mTOR Inhibition Reverts the Mettl8‐Mediated Neuronal Differentiation of NSCs

To establish a causal role for mTOR signaling in Mettl8‐regulated differentiation, we assessed the neuronal marker βIII‐Tubulin in the presence of rapamycin in rat NSCs. WB analysis showed that the increases in p‐mTOR, p‐4E‐BP1, and βIII‐Tubulin protein levels induced by sh‐Mettl8 were all abolished by rapamycin co‐treatment (Figure 6A–D). Immunofluorescence quantification further confirmed that sh‐Mettl8 significantly increased the proportion of βIII‐Tubulin+ neurons (17.1% ± 1.2%, *p* < 0.001), and this increase was completely suppressed by rapamycin (Figure 6E,F). This bidirectional rescue demonstrates that Mettl8 loss drives neuronal differentiation via the mTOR/4E‐BP1 axis in a pharmacologically reversible manner.

## 4 Discussion

Global aging has escalated neurodegenerative diseases into a public health crisis. With AD affecting 10% of individuals over 65 and projected to reach 150 million cases by 2050 [21], current therapies merely delay symptoms without reversing hippocampal neuronal loss. This study identifies the RNA methyltransferase METTL8 as a key negative regulator of hippocampal NSCs’ fates by suppressing the mTOR/4E‐BP1 axis. Expression profiling showed METTL8 enrichment in differentiated cells: qRT‐PCR revealed ≥ 4.5‐fold higher mRNA in neurons/astrocytes (*p* < 0.001), while WB confirmed 2.5‐fold and 1.7‐fold increased protein in neurons and astrocytes vs. NSCs (*p* < 0.05) (Figure 1), indicating its role in cell identity transition. Functionally, Mettl8 knockdown induced G0/G1 arrest (34.0% ± 1.3% increase, *p* < 0.001) with Cyclin E downregulation (1.8‐fold decrease, *p* < 0.05) and reduced EdU+ cells (*p* < 0.01) (Figure 2), consistent with “G1 lengthening promotes neurogenic division” [22, 23, 24]. Conversely, Mettl8 overexpression accelerated G1/S transition (8.9% ± 0.6% S‐phase increase, *p* < 0.01) and upregulated Cyclin E (Figure 2). Differentiation assays revealed METTL8's bidirectional control: Its loss elevated neuronal (19.7% ± 5.5% more βIII‐Tubulin+ cells, *p* < 0.05) and astrocytic markers (8.3% ± 1.6% more GFAP+ cells, *p* < 0.01) (Figure 3), highlighting its stemness maintenance role. Mechanistically, transcriptomics identified PI3K‐AKT–mTOR as the top‐enriched pathway among 1224 DEGs (Figure 4). Subsequent experiments confirmed METTL8‐mediated suppression of AKT (44.2%↓ phosphorylation, *p* < 0.05), mTOR (60.6%↓ phosphorylation, *p* < 0.01), and 4E‐BP1 phosphorylation (42.1%↓, *p* < 0.05) (Figure 5). Rapamycin (20 μM) rescued sh‐Mettl8‐induced neuronal differentiation (βIII‐Tubulin+ suppression, *p* < 0.001) (Figure 6), validating the cascade. Collectively, our findings identify METTL8 as a novel epitranscriptomic regulator involved in controlling the balance between NSCs’ proliferation and differentiation by inhibiting the mTOR/4E‐BP1 axis.

A seemingly paradoxical observation is that Mettl8 knockdown simultaneously activates the mTOR pathway and inhibits proliferation. This apparent contradiction can be resolved by considering the context‐dependent and biphasic nature of mTOR signaling in stem cell fate determination. While moderate mTOR activity is known to promote cell growth and cycle progression, sustained or excessive mTOR activation can trigger alternative programs such as differentiation, autophagy, or cell cycle exit [25, 26, 27]. In our model, loss of Mettl8 leads to a dysregulated, heightened activation of mTOR, which may exceed the threshold conducive to self‐renewal, as observed in other contexts of mTOR hyperactivation [27]. This aberrant signaling likely upregulates p‐4E‐BP1, reprogramming the translatome to favor the synthesis of proteins involved in differentiation and cell cycle arrest, thereby explaining the observed G1 lengthening and reduced Cyclin E. Thus, Mettl8 does not merely act as an “off switch” for mTOR; rather, it functions as a homeostatic tuner, maintaining mTOR activity within an optimal range that preserves the proliferative capacity of NSCs and prevents premature differentiation.

Our study contributes to the understanding of hippocampal neurogenesis by defining a previously unknown role for METTL8. First, we identify a novel function for METTL8. While METTL8 has been studied in other contexts (e.g., via STAT3 signaling) [13], its specific function as a regulator of neural stem cell cycle progression and differentiation has not been previously reported. Second, our findings offer an alternative perspective on the regulation of the PI3K‐Akt–mTOR pathway in fate determination. Contrasting the classic paradigm where growth factors (e.g., BDNF/IGF1) activate mTOR to promote regeneration and proliferation [28, 29], we demonstrate that METTL8 acts as an endogenous inhibitor of mTOR phosphorylation (Figure 5). This inhibition lengthens the G1 phase (Figure 2), thereby favoring differentiation—a finding that provides a direct molecular mechanism for the longstanding “cell cycle duration determines cell fate” hypothesis [22, 23, 24] and validates prior pharmacological observations [30, 31, 32]. Third, our work bridges disparate fields by establishing a direct link between RNA methylation (an epitranscriptomic mechanism), translational control (via 4E‐BP1), and core cell cycle machinery, suggesting a potential link between RNA methylation, translational control, and cell cycle machinery in neurogenesis. Finally, these insights have clear translational relevance. The pharmacological rescue using rapamycin (Figure 6) proves the druggability of the METTL8‐mTOR axis for fine‐tuning neurogenesis. Given the documented role of mTOR modulation in cognitive disorders [33], METTL8 represents a potential novel target for neurodegenerative therapy, potentially offering a more specific intervention point than broad mTOR inhibition [34].

Although the chain of evidence is complete, the following issues still need to be explored in depth. Regarding model validity, in vitro NSCs models lack critical in vivo microenvironmental cues (e.g., vascular signaling, microglial crosstalk); we propose developing 3D organoids incorporating cerebrovascular units [35] or applying in vivo two‐photon imaging to track hippocampal neurogenesis in conditional knockout mice [36]. Molecularly undefined substrate specificity requires CRISPR‐assisted RNA pulldown for target identification and cryo‐EM structural determination of Mettl8‐Eif4ebp1 complexes. Additionally, 4E‐BP1‐mediated translatome reprogramming (e.g., ribosome‐bound mRNA profiles) awaits exploration, and this may determine the direction of neuron differentiation [37]. For clinical translation, 20 μM rapamycin's off‐target autophagy risks [38, 39, 40] warrant designing brain‐penetrant allosteric inhibitors. Population heterogeneity demands establishing stratified iPSC‐NSCs biobanks from AD patients to evaluate the potential for clinical transformation. Furthermore, the theoretical contradictions are not fully explained. The significance of METTL8 enrichment in mature neurons remains unclear. Does it regulate synaptic plasticity? Or provide feedback control against excessive regeneration? Single‐cell and spatial transcriptomics could clarify these.

In conclusion, this study systematically demonstrates that Mettl8 plays a central role in maintaining hippocampal NSCs' proliferative homeostasis and suppressing premature differentiation. Consistent with a model of homeostatic tuning, Mettl8 achieves this by fine‐tuning the activity of the mTOR/4E‐BP1 signaling pathway. These findings provide a novel therapeutic axis for regulating neuroregeneration. Future studies should employ conditional knockout models and patient‐derived iPSCs to validate the therapeutic potential of modulating the Mettl8‐mTOR axis in neurodegenerative diseases, ultimately facilitating the clinical translation of chronologically precise intervention strategies.

## Author Contributions

Jiamin Xu, Liping Xu, Yang Li, Xuejian Kong, and Wenhui Ma performed the experiments. Jiamin Xu and Liping Xu wrote the manuscript. Yang Li and Linjie Xie analyzed the data. Ying Lyu and Aiguo Xuan designed, supervised, and revised the manuscript. All authors read and approved the final manuscript.

## Funding

This work was supported by the open research funds from the Sixth Affiliated Hospital of Guangzhou Medical University, Qingyuan People's Hospital, No. 202201‐207 (to AGX); Guangdong Basic and Applied Basic Research Foundation, Nos. 2023A1515010292 (to AGX), 2022A1515111185 (to YLyu); and the Research Ability Enhancement Project of Guangzhou Medical University, Nos. 02‐410‐2405067 (to YLyu), 02‐410‐2405140 and 2024XK03 (to LPX).

## Ethics Statement

All animal experiments were approved by the Institutional Animal Care and Use Committee of Guangzhou Medical University (S2022‐245) and were conducted in accordance with the national guidelines for animal welfare.

## Conflicts of Interest

The authors declare no conflicts of interest.

## Data Availability

The data that support the findings of this study are available from the corresponding author upon reasonable request.
